# P-96. Efficacy and Safety of *Lactobacillus reuteri* Supplementation Combined with Triple Therapy for Eradicating *Helicobacter pylori*: An Updated Meta-Analysis of Randomized Controlled Trials

**DOI:** 10.1093/ofid/ofae631.303

**Published:** 2025-01-29

**Authors:** Omar Elkoumi, Ahmed Elkoumi, Mariam Khaled Elbairy, Amr Khaled Elnegiry, Ahmed Ghanem

**Affiliations:** Faculty of Medicine, Suez University, Suez, As Suways, Egypt; Faculty of Oral and Dental Medicine, Egyptian Russian University, Badr City, Al Qahirah, Egypt; Faculty of Medicine, Suez University, Suez, As Suways, Egypt; Faculty of Medicine, Suez University, Suez, As Suways, Egypt; Loma Linda University Medical Center- Murrieta, Murrieta, California

## Abstract

**Background:**

The efficacy of Helicobacter pylori (H. pylori) triple therapy is declining over time. Lactobacillus reuteri (L. reuteri) is a probiotic that has shown promising results for eradicating H. pylori and decreasing adverse events. We aim to assess the efficacy of adding L. reuteri to the standard triple therapy for H. pylori eradication.

**Methods:**

We systematically searched PubMed, Embase, Cochrane Central, Web of Science (WOS), and Scopus databases from inception until May 2^nd^, 2024. We included any randomized controlled trial comparing individuals receiving viable L. reuteri or a placebo preceding, during, or after the standard triple therapy for H. pylori eradication. We used Review Manager 5.4 for all statistical analyses.

**Results:**

Seven randomized controlled trials with a total of 518 patients were included in the meta-analysis. We found that patients who received L. reuteri added to their triple therapy showed a higher eradication rate than those with triple therapy alone (HR: 1.16, 95% CI [1.03, 1.31], p = 0.01). The pooled results showed that the addition of L. reuteri to the triple therapy resulted in a significantly lower risk of taste distortion (RR: 0.59, 95% CI [0.39, 0.90], p = 0.01), diarrhea (HR: 0.44, 95% CI [0.28, 0.71], p = 0.0007), and constipation (HR: 0.52, 95% CI [0.31, 0.87], p = 0.01). There was no difference between both groups in experiencing epigastric pain (HR: 0.62, 95% CI [0.29, 1.33], p = 0.22), or nausea/vomiting (HR: 0.61, 95% CI [0.16, 2.40], p = 0.48).

**Conclusion:**

In contrast to a previous meta-analysis, L. reutri supplementation improved the H. pylori eradication rate. Adding L. reuteri to the triple therapy decreased diarrhea, constipation, and taste distortion compared to the standard triple therapy alone. To reinforce our conclusions, further clinical trials with larger sample sizes are required.

**Disclosures:**

**All Authors**: No reported disclosures

